# Supplementary material for: Surface Antifouling Modification on Polyethylene Filtration Membranes by Plasma Polymerization
Source: Materials (Basel). 2020 Nov 6;13(21):5020. doi: 10.3390/ma13215020 (PMC7664414; doi:10.3390/ma13215020)
Supplement: Supplementary file 1 [file materials-13-05020-s001.pdf]

materials

Supplementary

Surface Antifouling Modification on Polyethylene Filtration Membranes by Plasma Polymerization

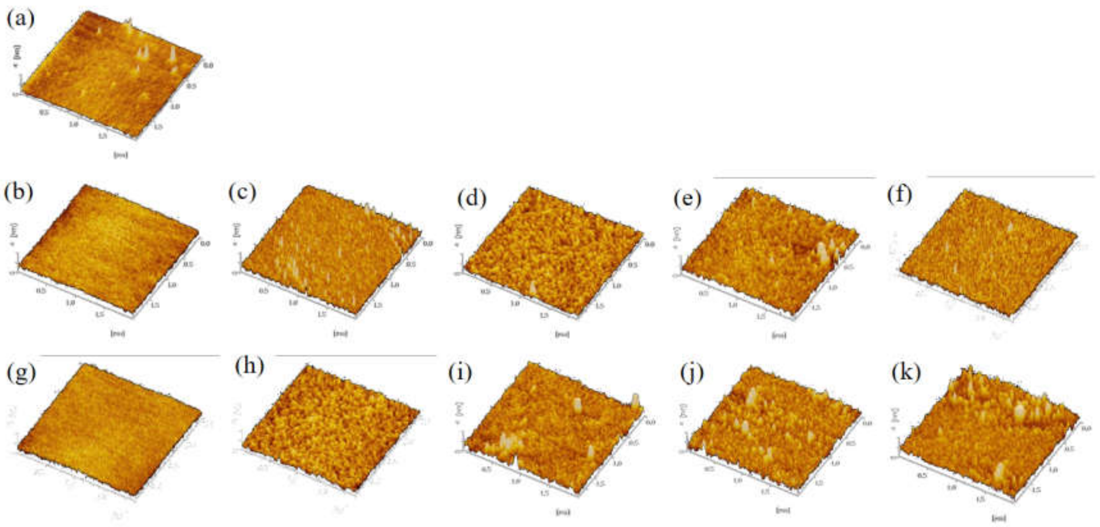

Figure S1. The AFM images on pristine PE (**a**), on pp-EO1V/Si at different applied plasma power: (**b**) 10 W, (**c**) 20W, (**d**) 30 W, (**e**) 40 W, (**f**) 50 W; and on pp-EO2V/Si at different applied plasma power: (**g**) 10 W, (**h**) 20W, (**i**) 30 W, (**j**) 40 W, (**k**) 50 W. (Plasma deposition pressure: 100 mtorr, flow rate of precursors: 15 sccm, deposition time: 0.5 h).

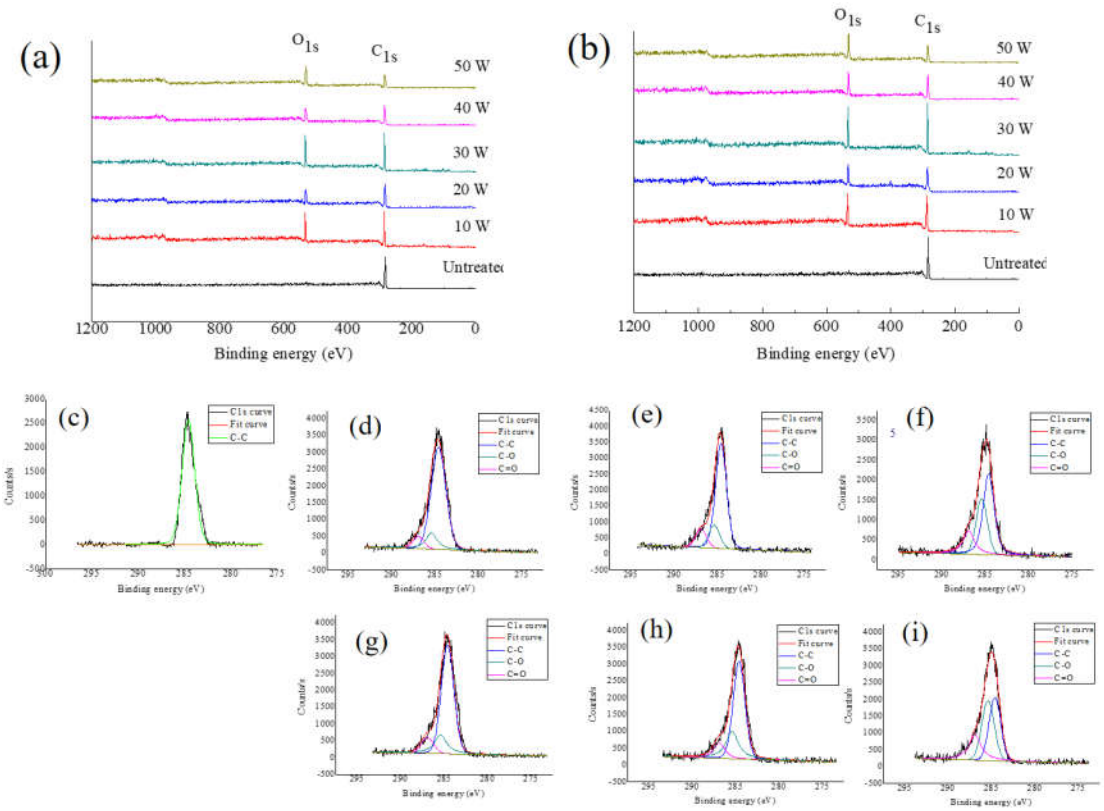

Figure S2. Wide scan ESCA spectra for the surface chemical composition as a function of the applied plasma power on (**a**) pp-EO1V/PE, and (**b**) pp-EO2V/PE. The high resolution C1s spectra on (**c**) pristine PE, (**d**) 10- pp-EO1V/PE, (**e**) 30-pp-EO1V/PE, (**f**) 50-pp-EO1V/PE, (**g**) 10-pp-EO2V/PE, (**h**) 30-pp-EO2V/PE, and (**i**) 50-pp-EO2V/PE. (Plasma deposition pressure: 100 mtorr, flow rate of precursors: 15 sccm, deposition time: 0.5 h).

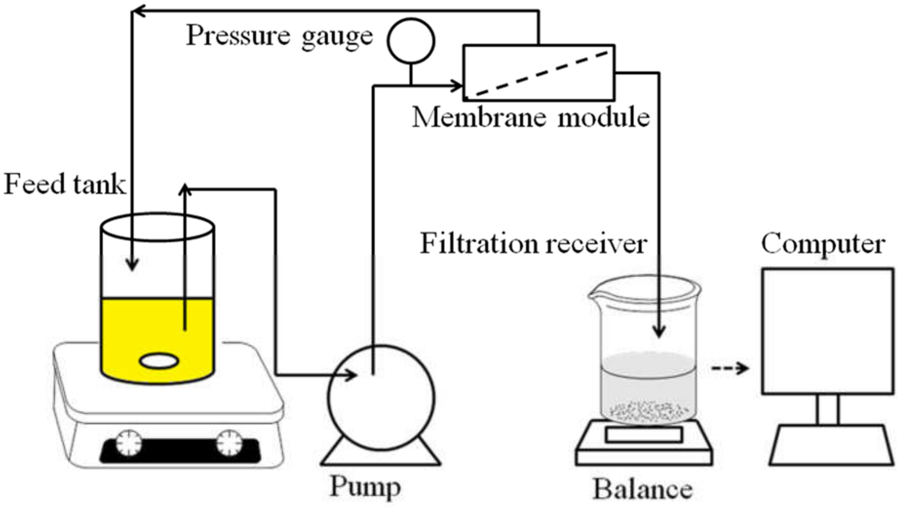

Figure S3. The set-up for measurement for filtration performance. The feed tank is used to reserve cell solutions; the tested PE membranes were incorporated into the membrane module. The filtrate performance was evaluated by weighting the cumulative weight of the filtrate cakes.

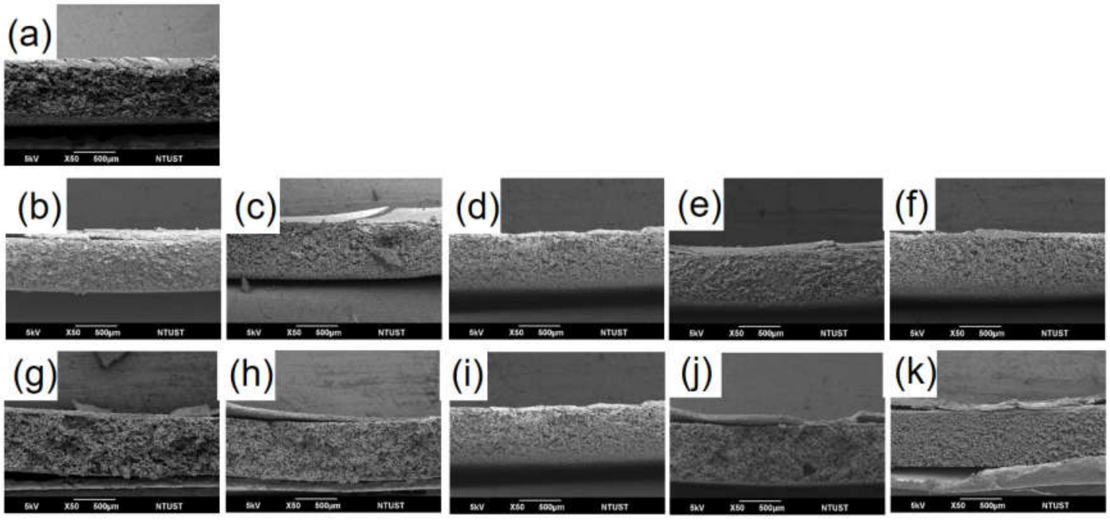

Figure S4. The thickness of filtrate cake after filtration of CHO cells on (**a**) pristine PE, (**b**) 10-pp-EO1V/PE, (**c**) 20-pp-EO1V/PE, (**d**) 30-pp-EO1V/PE, (**e**) 40-pp-EO1V/PE, (**f**) 50-pp-EO1V/PE, and (**g**) 10-pp-EO2V/PE, (**h**) 20-pp-EO2V/PE, (**i**) 30-pp-EO2V/PE, (**j**) 40-pp-EO2V/PE, (**k**) 50-pp-EO2V/PE.
